# 3D printable shape-customized hydrogel thermocells

**DOI:** 10.1093/nsr/nwaf518

**Published:** 2025-11-19

**Authors:** Lili Liu, Yiwen Bo, Ding Zhang, Jiaqi Guo, Wenqi Fan, Ruohan Niu, Xin Guo, Xiaotian Jiang, Rujun Ma

**Affiliations:** School of Materials Science and Engineering, Nankai University, Tianjin 300350, China; School of Materials Science and Engineering, Nankai University, Tianjin 300350, China; School of Materials Science and Engineering, Nankai University, Tianjin 300350, China; School of Materials Science and Engineering, Nankai University, Tianjin 300350, China; School of Materials Science and Engineering, Nankai University, Tianjin 300350, China; School of Materials Science and Engineering, Nankai University, Tianjin 300350, China; School of Materials Science and Engineering, Nankai University, Tianjin 300350, China; School of Materials Science and Engineering, Nankai University, Tianjin 300350, China; School of Materials Science and Engineering, Nankai University, Tianjin 300350, China

**Keywords:** 3D printing, hydrogel thermocells, customized design, maximized heat utilization, thermoelectric conversion

## Abstract

Hydrogel thermocells based on the thermogalvanic effect hold significant promise for energy harvesting and flexible electronics due to their excellent heat-to-electric conversion and stretchability. However, mismatched contact surfaces between bulk hydrogel thermocells and complex heat-source geometries often lead to poor heat utilization and reduced conversion efficiency. While 3D printing can create structures that match these complex interfaces, direct printing of hydrogel thermocells remains challenging due to issues such as water evaporation and the incompatibility of redox couples with photopolymer inks. To overcome these challenges, we combine 3D digital light processing with a thermoelectric (TE) solvent-exchange strategy, enabling the precise customization of stretchable hydrogel thermocells (DHFGs) that conform to specific heat-source geometries. Nonvolatile deep eutectic solvents in the photopolymer ink precursors form robust eutectogel networks through ultraviolet-initiated polymerization, ensuring high structural fidelity (down to 130 μm). The resulting 3D-printed thermocells exhibit excellent TE performance (3.5 mV K^−1^) and can be tailored to complex geometries. This conformal design extends the effective working ambient temperature range by 6.0 K and boosts output power to ∼350% of that of an unmatched DHFG. Additionally, the microstructured DHFG demonstrates superior pressure sensitivity (0.35 kPa^−1^) within a low-pressure range (<1.0 kPa), at ∼4.4 times higher than its unstructured counterparts. This approach optimizes thermal-energy utilization through geometric matching, simplifies assembly and paves the way for flexible thermocells with high TE performance, complex architectures and multifunctionality.

## Teaser

This work introduces a 3D-printed, shape-customized hydrogel thermocell that maximizes heat utilization through precise geometric matching, achieving high thermoelectric performance and multifunctionality for next-generation wearable energy devices.

## INTRODUCTION

Flexible thermoelectric (TE) materials and devices provide a promising platform for flexible power supplies, self-powered systems and flexible sensors by efficiently harvesting low-grade thermal energy [1–5]. Among them, hydrogel-based thermocells have garnered significant attention due to their remarkable flexibility, stretchability, leak-proof properties, high Seebeck coefficient (*S*_c_) and outstanding energy-conversion efficiency [1,2,6–11]. For hydrogel thermocells, thermal-energy harvesting is governed by two primary mechanisms. The first mechanism relies on the Soret effect, in which ionic thermodiffusion is driven by a temperature gradient [9]. However, this effect is inherently limited by the equilibrium between ion migration within the hydrogel electrolyte and electron migration in the external circuit, thus limiting the continuous electrical output under a steady temperature difference (Δ*T*) [12]. In contrast, the second mechanism, namely the thermogalvanic effect, utilizes redox reactions occurring at electrodes maintained at different temperatures, allowing thermocells to sustain electricity generation. Redox couples commonly used in these systems include iron/ferricyanide ([Fe(CN)_6_]^4^^−^^/3−^), iodide/triiodide and iron (II/III) [7,10]. Moreover, the incorporation of additives such as guanidine and amide derivatives has been shown to significantly increase the entropy change (Δ*S*) of the system, thereby enhancing the *S*_c_ and overall TE performance [8].

Despite their promise, existing fabrication methods for hydrogel thermogalvanic thermocells, including immersion [13–15], *in situ* polymerization [14] and sol–gel methods [16], typically yield relatively monolithic structures. These approaches cannot effectively address the complex geometries of natural and industrial heat sources, such as those found in hot-spring vents, engine blocks, exhaust-pipe joints and water-pipe bends. The mismatch between the surface of the hydrogel thermocell and the geometry of the irregular heat sources leads to a significant reduction in heat utilization and thus a drastic decrease in the heat-to-electricity conversion efficiency [17–19]. To address this limitation, it is critical to design hydrogel thermocells that can be perfectly matched to these complex heat-source geometries in order to maximize the thermal-energy-conversion efficiency.

The rise of 3D printing technologies has revolutionized fields such as biomedical engineering and flexible electronics, owing to their ability to precisely customize structures for rapid and high-resolution prototyping [18,20–25]. Combining 3D printing technology with hydrogel thermocells offers a unique opportunity to enhance heat utilization and energy-conversion efficiency. Among various 3D-printing methods, extrusion-based direct ink writing (DIW) technology is promising for producing customized hydrogel structures for biomedical applications [26,27]. However, DIW suffers from limitations such as low resolution, slow printing speed and material deformation, leading to a time-consuming printing process [28,29]. In addition, the stringent requirements for ink viscosity and solubility further limit its applicability.

In contrast, digital light processing (DLP) offers notable advantages, including higher resolution and faster printing speeds, making it particularly well suited for fabricating hydrogel thermocells. In DLP-3D printing, organogels with low-volatility solvents, such as ionic liquids and deep eutectic solvents (DESs), have emerged as an ideal alternative to water-based systems [21,22,30]. DESs, formed from hydrogen-bond donors and acceptors, has excellent ionic conductivity, stability, nontoxicity and nonvolatility, making it an ideal choice for high-performance organogels in 3D-printing applications [21,31–33]. However, the fabrication of hydrogel thermocells based on DLP technology still faces several challenges, including structural deformation due to water evaporation and incompatibility between common redox pairs and photopolymerization ink precursors [34–37]. To address these challenges, combining DES-based 3D printing with the thermogalvanic effect provides an effective strategy for precisely customizing hydrogel thermocells with complex structures that are adapted to the heat sources, thereby optimizing heat utilization.

Building on the above analysis, a DLP-based eutectogel (DH) with a dual network structure was developed by utilizing a DES and the monomer 2-hydroxyethyl acrylate (HEA). This approach resulted in the fabrication of a customized high-resolution flexible thermocell (DHFG) through the subsequent [Fe(CN)_6_]^4^^−^^/3−^ solvent exchange. The incorporation of nonvolatile DES and HEA into the photopolymer ink precursor facilitated the formation of a robust DHFG polymer network with excellent structural stability and resolution. The optimized 3D-printed DHFG demonstrated exceptional thermopower performance of 3.5 mV K^−1^ with high structural resolution (down to 130 μm). By tailoring the DHFG to match complex heat-source geometries, the resulting thermocells output is almost 350% of that of the mismatched DHFG, while effectively extending the effective working ambient temperature range by 6.0 K. Furthermore, the microstructured DHFG exhibited a remarkable pressure sensitivity of 0.35 kPa^−1^ below 1.0 kPa, which is nearly 4.4 times higher than that of the unstructured DHFG (0.08 kPa^−1^), highlighting their potential for motion-sensing and health-monitoring applications. This work demonstrates significant advances in the fabrication of flexible thermocells with high TE performance, precise structural customizability and high resolution. These advancements enhance adaptability to a variety of heat sources, enabling efficient thermal-energy conversion and multifunctional wearable applications, and paving the way for the broader adoption of next-generation TE technologies.

## RESULTS AND DISCUSSION

### 3D printing of DHFG

**Figure 1.**
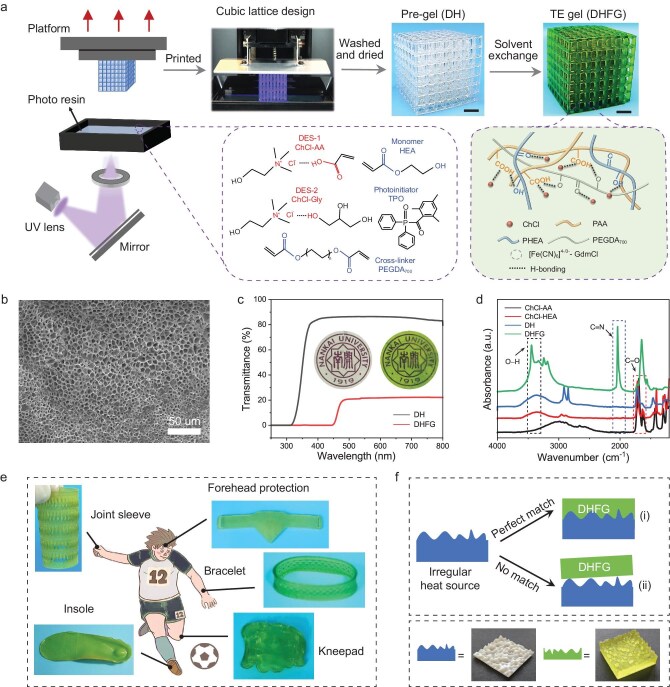
Fabrication and characterization analysis of 3D-printed customized DHFG wearable thermocell. (a) Preparation process of high-fidelity photopolymerization 3D-printed cubic lattice structure of the DHFG. Multiple hydrogen bonds interact within the DHFG. The scale bar is 1 cm. (b) Scanning electron microscopy image of the DH. (c) Transmittance spectra of the DH and DHFG, with insets illustrating the high degree of transparency of the DH. (d) Fourier transform infrared spectra of ChCl–AA, ChCl–HEA, DH and DHFG. (e) Customized DHFG images of wearable finger-joint sleeves, bracelets, insoles, knee joints and headbands for body parts. (f) (i) Perfect match formed by the irregular heat source and the 3D-printed microstructured DHFG and (ii) mismatch of the unstructured DHFG.

Figure 1a shows the DHFG with a cubic structure fabricated by using 3D printing (see ‘Methods’ for details). Briefly, the photopolymer ink comprises monomers of HEA and acrylic acid (AA), a cross-linking agent of poly (ethylene glycol) diacrylate (PEGDA_700_), a water-soluble photoinitiator made of diphenyl (2,4,6-trimethyl benzoyl) phosphine oxide (TPO) and a nonvolatile DES. The DES, as the precursor solvent, consists of choline chloride–acrylic acid (ChCl–AA, DES-1) and choline chloride–glycerol (ChCl–Gly, DES-2), which can be used to promote the formation of stable eutectogel networks by ultraviolet-curing polymerization. Among them, ChCl acts as a hydrogen-bond acceptor and AA as a hydrogen-bond donor. Gly further reduces the solvent viscosity and enhances the flexibility of polymer chains. In addition, the poly (N-hydroxyethyl acrylamide) (PHEA) forms a double-network scaffold with the ChCl/AA supramolecular deep eutectic polymer network. PHEA molecular chains, rich in hydroxyl and carbonyl groups, provide ample hydrogen-bonding sites, reinforcing the non-covalent interactions between molecular chains.

PEGDA_700_ covalently links the ionic monomers to endow the gel with excellent tensile properties. A homogeneous solution for 3D printing (D_a_H_b_, where a and b represent the mass ratios of the DES and HEA, respectively) is achieved by mixing varying amounts of HEA with the DES (Fig. S1a). After printing, the DH was immersed in ethanol to remove unreacted monomers and solvents, followed by drying in an oven at 60°C for 30 minutes. This drying step is crucial for eliminating residual ethanol, thus preventing its potential dilution or contamination effect on the subsequent TE solution. The dried DH was then soaked in a solution containing the [Fe(CN)_6_]^4^^−^^/3−^–guanidinium chloride (GdmCl) at 80°C for 3 hours to obtain the hydrogel thermocell, denoted as DHFG. Meanwhile, the utilization of DES could maintain the original 3D structure of the supramolecular deep eutectic polymer network after immersion in the TE solution. The DHFG exhibits excellent elasticity and adapts well to bending, twisting and stretching (Fig. S1b).

The scanning electron microscopy image shows that the DH gel has a characteristic porous microstructure (Fig. 1b). Figure 1c shows the high transparency (80%) of the DH in the visible-light range, whereas, after TE immersion treatment, the transparency of the DHFG is reduced to 20%, demonstrating that redox ions are well bound to the gel. The interactions and structural changes within the printed DH network were further analysed and investigated by using Fourier transform infrared spectroscopy (FTIR). As shown in Fig. 1d, the broad peak at 3030 cm^−1^ corresponds to the O–H stretching vibration in the AA [32]. With the progressive addition of each element, a broad peak appears in the range of 3200∼3700 cm^−1^. Additionally, the increased non-covalent interaction between components enhances the characteristic absorption peak at 3300 cm^−1^, which is attributed to O–H stretching vibration in the HEA [32]. The C=O vibration peak shifts from 1694 cm^−1^ in the ChCl–AA to 1716 cm^−1^ in the DH, alongside a redshift in the O–H stretching vibration, confirming hydrogen-bonding interactions between the HEA and AA chains [21,33]. Furthermore, the C≡N tensile vibration observed at 2040 cm^−1^ in the DHFG indicates the successful solvent exchange with the [Fe(CN)_6_]^4^^−^^/3−^–GdmCl solution [11]. Fig. S2 further shows the FTIR spectrum of the optimized thermocell, in which the increase in the HEA content amplifies the O–H tensile vibration peak, highlighting the stronger interaction between the HEA and the DES [32]. Prominent vibration peaks at C≡N also confirm the completion of the TE solvent exchange. These results indicate that the DHFG is rich in hydrogen bonding, which provides dynamic cross-linking sites that serve as a ‘sacrifice domain’ for mechanical energy dissipation during stretching, thereby enhancing its mechanical properties.

These structural and mechanical features endow the 3D-printed DHFG with strong adaptability to complex, real-world thermal environments. As shown in Fig. 1e, using the human body as the heat source, 3D-printing technology was employed to customize wearable DHF_0.2_G_3_ structures suitable for different parts of the human body, including finger-joint sleeves, bracelets, insoles, knee joints and headbands. This customization is pivotal to our exploration of how the structural design of wearable thermocells influences thermal-energy harvesting efficiency. To achieve effective contact matching with complex heat sources (simulated here by a material with an irregularly shaped interface, as illustrated in Fig. 1f), one side of the DHF_0.2_G_3_ was meticulously engineered via 3D printing to precisely align with this irregular heat surface. When the irregular surface of the printed DHF_0.2_G_3_ comes into contact with the heat source, a perfect match occurs; in contrast, the smooth surface of the DHF_0.2_G_3_ will result in a mismatch. These two contact scenarios, which directly impact the thermal-energy-collection efficiency, will be analysed in subsequent sections to elucidate the role of customized 3D-printed structures in optimizing wearable thermocell performance.

### Resolution and mechanical properties of DHFG

3D printing is performed by using layer-by-layer lithography, which requires photocurable inks with photosensitivity and low viscosity [23,32,34]. In this study, we kept a constant ratio of the hydrogen-bond donor to the acceptor in the DES and focused on the effect of the DES-to-HEA ratio on ink viscosity. The specific gel formulation, D_a_H_b_, utilized TPO as a photoinitiator and PEGDA_700_ as a cross-linking agent to initiate the photopolymerization reaction (a and b represent the mass ratios of the DES and HEA, respectively). Incorporating the HEA can improve the 3D-printing properties, including printing costs, at a high resolution of 130 μm (see Fig. S3 for details).

**Figure 2.**
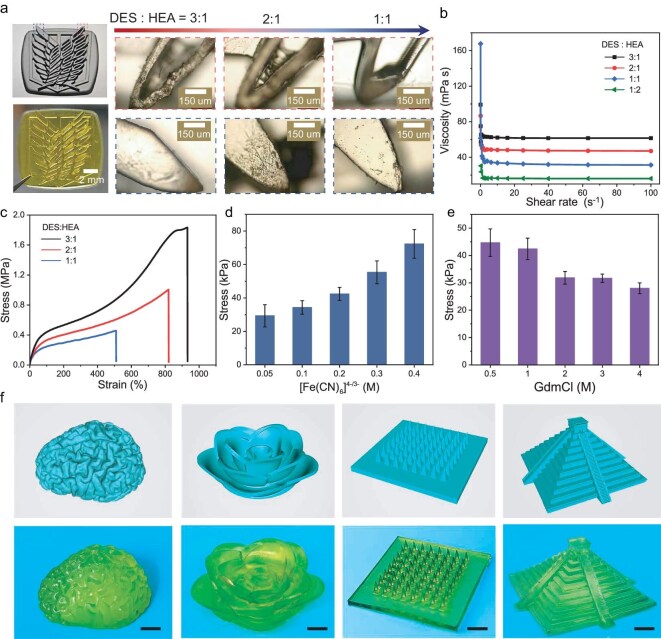
Resolution testing and mechanical properties of 3D-printed objects. (a) Digital image of the stereolithography file and photograph from the resolution tests of 3D-printed D_a_H_b_ using DES: HEA at mass ratios of 3:1, 2:1 and 1:1. The resolution of the three printed samples was evaluated by magnifying two regions containing alternating grooves and plateaus under a microscope. (b) Shear-thinning characteristics of the D_3_H_1_, D_2_H_1_, D_1_H_1_ and D_1_H_2_ formulations. (c–e) Mechanical performance of (c) D_3_H_1_, D_2_H_1_ and D_1_H_1_, (d) the DHF*_x_*G_1_ with 1.0 M of GdmCl (*x* ranging from 0.05 to 0.4 M), where the *x*-axis represents the concentration (*x*) of [Fe(CN)_6_]^4^^−^^/3−^, and (e) the DHF_0.2_G*_y_* with 0.2 M of [Fe(CN)_6_]^4^^−^^/3−^ (*y* from 0.5 to 4.0 M), where the *x*-axis represents the concentration (*y*) of GdmCl. (f) CAD models and the printed constructions of various structures, including a cerebrum, flower, microneedles and tower. The scale bar is 1 cm.

Figure 2a shows microscopic images and photos of stereolithography files printed with the DES and HEA at mass ratios of 3:1, 2:1 and 1:1. The resolution test on the 3D-printed samples involved magnifying alternating grooves and protrusions under a microscope. While the D_3_H_1_, D_2_H_1_ and D_1_H_1_ formulations accurately replicated the structure, D_3_H_1_ exhibited partial precursor curing in the sample tank due to implosion during printing, increasing the printing costs. Meanwhile, the D_1_H_1_ formulation showed minor structural distortion in the grooves due to insufficient resolution and a low modulus. In contrast, D_2_H_1_ produced a well-defined structure with smooth surfaces and sharp edges, closely matching the predicted dimensions without losing raw materials. Printability assessments showed that D_1_H_2_ failed to print effectively due to its low modulus. These findings highlight that the DES can significantly enhance the polymerization rate within eutectic solvents through multiple hydrogen and ionic bonds. Moreover, an increased DES concentration can drastically accelerate the polymerization process, resulting in faster gelation. Based on these observations, D_2_H_1_ was selected as the optimal printing ratio for our subsequent work.

To be compatible with 3D printing, photocurable inks must have low-viscosity fluidity [32]. Figure 2b shows the shear-thinning tests for four printed ratios, with D_1_H_2_ displaying the lowest shear-thinning properties due to its stable network in the initial state. As the HEA content increases, the viscosity of the precursor liquid gradually decreases, indicating an incomplete monomer reaction. The D_3_H_1_ ink, characterized by strong hydrogen bonding between DES and HEA molecular chains, demonstrates the enhanced mechanical properties of the DH. The D_3_H_1_, D_2_H_1_ and D_1_H_1_ inks are low-viscosity fluids of <90 mPa, which meets the requirement for photocuring 3D printing [38]. Therefore, the DES and HEA ratio directly affects the stress–strain properties of the printed gel (Fig. 2c). By combining the HEA with a flexible poly(acrylic acid) (PAA) chain to form a double network (DN) structure, the HEA acts as a flexible network and breaks the gel when subjected to external forces. The HEA dissipates energy during the stretching process and is therefore considered a ‘sacrifice bond’. When greater stress is applied, the HEA in the HEA/PAA DN gel could break further and give the gel a certain strength and toughness. For instance, the elongation of D_3_H_1_ and D_2_H_1_ is >800% and the breaking strengths are 1.0 and 1.8 MPa, respectively, exhibiting elastomer-like mechanical properties. As the DES content decreases, the fracture strength of D_1_H_1_ decreases to 0.4 MPa. Figure 2d and e illustrates the fracture strength of printed DHF*_x_*G_1_ and DHF_0.2_G*_y_*, respectively (DH represents D_2_H_1_, similarly to below).

The mechanical strength of the DHFG increased from 29.3 kPa at *x* = 0.05 to 72.3 kPa (Fig. 2d) at *y* = 0.4 and from 28 kPa at *y* = 4 to 44.6 kPa at *y* = 0.5 (Fig. 2e). Due to the solvent exchange between the eutectic solvent and aqueous phases, the strength of the DHFG is lower than that of the DHs, but still does not affect the resolution of the print [39,40]. The interaction of the HEA and AA segments with the [Fe(CN)_6_]^4^^−^^/3−^ affects molecular chain aggregation and mechanical properties. On the one hand, the [Fe(CN)_6_]^4^^−^^/3−^ polarizes water molecules, forcing them to flow out between the polymer chains, leading to aggregation or crystallization of the HEA/AA chains (Fig. S4). As the concentration of the [Fe(CN)_6_]^4^^−^^/3−^ increases, the ‘salting out’ intensifies, resulting in a denser polymer chain and enhanced mechanical properties of DHF*_x_*G_1_ [41]. On the other hand, when GdmCl is added, Gdm^+^ interacts with the HEA/AA chain, creating a ‘salt in’ effect. Gdm^+^ destroys the hydrated shell around the [Fe(CN)_6_]^4^^−^^/3−^ complex, restores the hydrogen bond between the HEA/AA and its hydrated water, and reduces the crystallinity (Fig. S5). Therefore, with the decrease in *y*, the intensity of SAF_0.2_G*_y_* increases. Moreover, the antifreezing performance of the DHFG is tuned by varying the extent of the solvent displacement. While the TE and mechanical properties were optimized in collaboration, the DHF_0.2_G_3_ obtained at 80°C for 3 h showed excellent antifreeze ability at –48°C through the differential scanning calorimetry measurement (Fig. S6).

Additionally, the versatility of the 3D-printed DH enables the fabrication of complex DHFG architectures such as cerebral structures, flowers, microneedles and towers with high fidelity and at sub-millimeter feature resolution (Fig. 2f). Although the achieved printing resolution of 130 μm represents a significant improvement over those of conventional extrusion-based methods for hydrogel thermocell fabrication, it still presents limitations for applications requiring extreme miniaturization, such as high-density microfluidic systems or neural interfaces. To further enhance manufacturing precision and broaden the application scope, future efforts will focus on integrating high-resolution 3D-printing techniques, such as two-photon polymerization and advanced DLP systems, which can produce micron- or submicron-scale features. In parallel, we will optimize photopolymer ink by introducing highly efficient and wavelength-stable photoinitiators. In addition, careful adjustment of the exposure conditions and monomer composition will be carried out to further enhance printing precision and device performance.

### Optimization and mechanism of TE properties of DHFG

**Figure 3.**
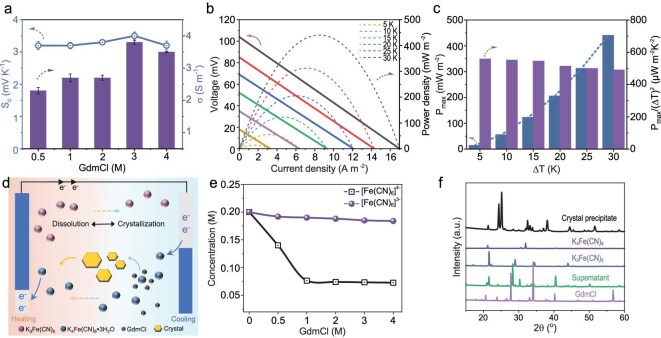
TE properties and mechanism of the DHFG. (a) *S*_c_ and *σ* of DHF_0.2_G*_y_* with 0.2 M of [Fe(CN)_6_]^4^^−^^/3−^ (*y* ranging from 0.5 to 4.0 M), where the *x*-axis represents the concentration (*y*) of GdmCl. (b) Output voltage–current density curves and (c) maximum *P*_max_/(Δ*T*)^2^ of the DHF_0.2_G_3_ under different Δ*T* values. (d) Schematic of the role of GdmCl in enhancing the thermogalvanic effect in DHF_0.2_G_3_. (e) Concentration changes in [Fe(CN)_6_]^4−^ and [Fe(CN)_6_]^3^^−^ based on ultraviolet-visible spectroscopy (UV-vis spectra) in 0.2 M of [Fe(CN)_6_]^4^^−^^/3−^ and *y* M of GdmCl solution (y = 0, 0.5, 1.0, 2.0, 3.0 and 4.0). (f) X-ray diffraction spectroscopy (XRD spectra) of pure K_4_Fe(CN)_6_, GdmCl, K_3_Fe(CN)_6_, lyophilized precipitate and supernatant powders. Error bars represent standard deviations from at least three evaluations per sample.

The printed DH was immersed in a [Fe(CN)_6_]^4^^−^^/3−^–GdmCl solution to transform it into a high-fidelity 3D-printed DHFG. By optimizing the ratio of [Fe(CN)_6_]^4^^−^^/3−^ to GdmCl, the DHF_0.2_G_3_ achieved optimal *S*_c_ (3.5 mV K^−1^) and conductivity (*σ*) (3.8 S m^−1^), as depicted in Fig. 3a. The strong interaction potential of the GdmCl with the [Fe(CN)_6_]^4^^−^^/3−^ promoted the crystallization of the [Fe(CN)_6_]^4−^, which enhanced the TE performance (Fig. 3a and Figs S7 and S8). Comparative analysis of the power factor (PF) and figure of merit (ZT) showed that the DHF_0.2_G_3_ outperformed other formulations, achieving a PF of 46 µWm^−1^ K^−2^ and a ZT of 0.022 (Fig. S9). Even under 150% tensile strain, the DHF_0.2_G_3_ maintained a stable output voltage of 35.0 mV with a *S*_c_ of 3.5 mV K^−1^, as shown in Fig. S10a. In addition, the *S*_c_ of the DHF_0.2_G_3_ was tested at different cycles under 150% strain. As shown in Fig. S10b and c, there is no obvious variation in the mechanical stability and *S*_c_ during the entire test process. This further confirms that the *S*_c_ of the DHFG is mainly determined by the solvent–structure entropy difference and the difference in the concentration ratio of the redox pair, and is not affected by long-term cyclic stretching. The normalized output power density (*P*_max_/(∆*T*)^2^) for the DHF_0.2_G_3_ at ∆*T* ∼ 10 K was calculated at 551 µWm^−2^ K^−2^ (Fig. 3b and c, and Equation S1). Moreover, at Δ*T* ∼ 30 K, the thermal-energy conversion efficiency (*ƞ*) and Carnot relative efficiency (*ƞ*_r_) of the DHF_0.2_G_3_ reached 0.046% and 0.5%, respectively (Fig. S11 and Equations S2 and S3).

The incorporation of Gdm^+^ into the [Fe(CN)_6_]^4^^−^^/3−^ pair significantly enhances the TE performance of the DHFG, as illustrated in Fig. 3d. At a specific temperature difference (∆*T*), a redox reaction occurs at the hot and cold electrodes. The interaction between the electrode and the [Fe(CN)_6_]^4^^−^^/3−^ pair generates a potential difference, while the thermodynamically unstable concentration gradient is balanced. Gdm^+^ disrupts the hydration shell around the [Fe(CN)_6_]^4−^, triggers crystallization of the [Fe(CN)_6_]^3−^, and facilitates a reversible redox process. These crystals exhibit high thermal solubility, releasing [Fe(CN)_6_]^4−^ ions on the hot side, which increases the concentration, promotes oxidation and generates more [Fe(CN)_6_]^3−^ ions. These ions migrate to the cold side, further enhancing the reduction and boosting the thermopower [11,42]. The impact of the GdmCl on the concentrations of the [Fe(CN)_6_]^4^^−^ and [Fe(CN)_6_]^3−^ was validated via ultraviolet-visible spectroscopy (UV-vis spectra) (Fig. 3e and Figs S12 and S13). According to the Lambert–Beer law, the [Fe(CN)_6_]^4−^ concentration rapidly decreases from 0.2% to 0.08%—a more significant reduction than that observed for the [Fe(CN)_6_]^3−^, indicating that GdmCl interacts with the [Fe(CN)_6_]^4−^ to form a new precipitate [2,11]. The X-ray diffraction pattern (XRD pattern) (Fig. 3f) further confirms that Gdm^+^ triggers the precipitation of the [Fe(CN)_6_]^4−^, thus enhancing the redox reaction and improving the TE properties of the DHF_0.2_G_3_ [1].

### Design and performance of high-sensitivity DHFG sensor arrays

The ability to fabricate a DHFG of various shapes and configurations through 3D printing presents significant potential for developing flexible sensors with predesigned complex structures. Previous studies have reported that structural sensors with microstructures exhibited substantially enhanced sensitivity compared with nonstructural sensors, owing to their larger deformation under applied forces [43]. However, most microstructures are conventionally manufactured by using die transfer techniques [44], such as chemical etching or lithography, which are time-consuming and constrained by limited resolution. In contrast, 3D printing offers lower cost, higher production efficiency and enhanced precision, making it ideal for manufacturing flexible strain sensors [43,45,46].

**Figure 4.**
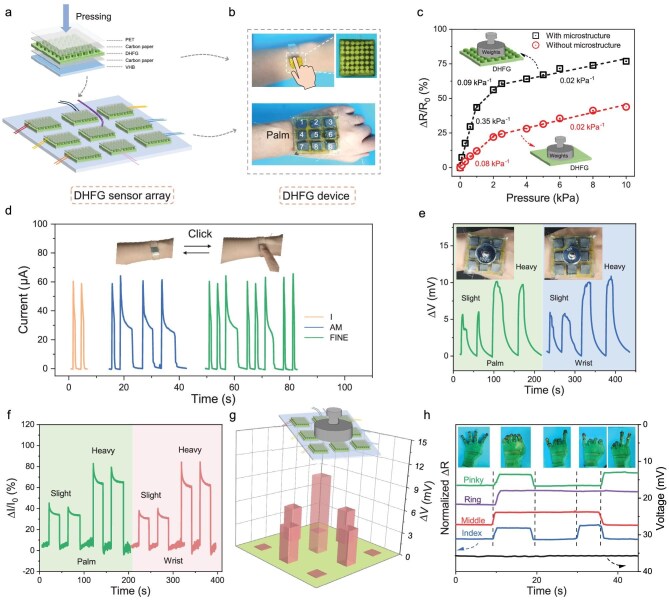
Design and performance of high-sensitivity DHFG sensor arrays. (a) Schematic and (b) optical images of a micrometer-scale cylinder DHF_0.2_G_3_ sensor and a 3 × 3 pixel DHF_0.2_G_3_ sensor array. (c) Relative resistance response of DHF_0.2_G_3_ sensor with and without cylinder when pressed by objects of varying forces. (d) Morse-code signal waveform of the DHF_0.2_G_3_ sensor wristband reading ‘I AM FINE’. (e, f) The DHF_0.2_G_3_ sensor array is placed on the human hand and wrist, monitoring the variation in (e) output voltage and (f) the relative current produced by objects with different pressures. The inset in (e) shows two weights positioned on the sensor array on the palm. (g) 3D bar charts depicting pressure distribution and the output voltage when an object is centered on the 3×3 pixel DHF_0.2_G_3_ sensor array. (h) Changes in normalized relative resistance and output voltage of the multiple sensors corresponding to the index, middle, ring and pinky fingers of the DHF_0.2_G_3_ manipulator for five gestures. The inset shows images of these gestures.

In this study, DHFG microstructured pressure sensors with micrometer-scale cylinder arrays were fabricated by using 3D printing. The dimensions of the sensors were 13 mm × 13 mm, with cylindrical microstructures with a height of 2.0 cm and diameter of 0.5 mm, and a clearance of 1.0 mm between the cylinders (Fig. 4a and b). Two types of DHFG sensors with and without microstructures (planar) were connected via copper wires to monitor the relative resistance change (Δ*R*/*R*_0_) under pressure stimuli. Here, *R*_0_ represents the initial resistance of the sensor in the absence of pressure and Δ*R* is the change in resistance under applied pressure, serving as a measure of sensor sensitivity. The sensitivity of the microstructured DHFG sensor was evaluated and compared with that of the planar sensor. Figure 4c shows the Δ*R*/*R*_0_ of the microstructured and unstructured DHFG under different pressures. Notably, the response curves revealed multiple linear regimes for applied pressure versus relative resistance change, with both exhibiting an increasing trend in resistance to change with rising pressure. In the low-pressure range (<1.0 kPa), the microstructured DHFG shows a faster growth rate of ∼0.35 kPa^−1^, nearly 4.4 times higher than that of the unstructured DHFG (0.08 kPa^−1^). As the pressure continues to increase, the growth rate of both becomes relatively smaller (0.02 kPa^−1^). This superior performance underscores the suitability of microstructured DHFG sensors for applications such as motion sensing and health monitoring (Fig. 4c).

Utilizing the exceptional temperature and current response properties of the DHF_0.2_G_3_, the effectiveness of the DHF_0.2_G_3_ in the communication of individuals with speech disabilities was explored. By integrating Morse coding, users could generate current signals of varying peak widths by touching the microstructured DHF_0.2_G_3_ surface. As shown in Fig. S14, brief (1-s) and prolonged (3-s) contact of the finger on the DHF_0.2_G_3_ produced distinct signals corresponding to the ‘dots’ and ‘lines’ in Morse code, respectively. Electrical signals based on the thermogalvanic effect confirmed the feasibility of encoding ‘0’ and ‘1’ signals by using this approach. A Morse-code table for A–Z is provided in Fig. S15. To demonstrate a practical application, the DHF_0.2_G_3_ sensor was integrated into a wristband, enabling individuals with aphasia to communicate effectively. For instance, the phrase ‘I AM FINE’ was translated into a corresponding waveform, providing a convenient communication tool (Fig. 4d).

To further explore the spatial-sensing capabilities, a 3×3 pixel DHF_0.2_G_3_ sensor array was developed and attached a wrist to create a self-powered information-conversion device for human–computer interaction (Fig. 4b and Figs S16 and S17). The spatial temperature and pressure-sensing performance of the array was systematically analysed. Initially, the DHF_0.2_G_3_ sensors attached to the wrist or palm captured various external stimuli (Fig. S18). As depicted in Fig. 4e and f, the output voltage and current signals quantitatively identified the stimuli when the DHF_0.2_G_3_ sensor array interacted with objects of varying temperatures or pressures. The output voltage and current signals varied with the applied pressure, ranging from 0.8 to 2.5 kPa. The peak voltage increased from 6 to 10 mV, while the relative current rose from 34% to 64%.

To examine the spatial-sensing capabilities, the center of the DHF_0.2_G_3_ sensor array was pressed with a force of 0.8 kPa. The spatial tactile information collected by the DHF_0.2_G_3_ sensor array was recorded (Fig. 4g). The spatial signal distribution of the temperature corresponded to the position of the object, linked to the contact area between the object and the pixel. The signal measured by the center pixel was more substantial than that measured by the surrounding pixels, with the four surrounding pixels exhibiting nearly the same contact area as the object. Figs S19 and S20 present voltage-mapping images and 3D bar charts showing the pressure distribution and relative current. Our findings suggest that DHFG sensor arrays can be tailored for personalized human–machine interfaces and information-encryption systems. The multifunctionality of the DHF_0.2_G_3_ manipulator was further validated by integrating it into a multichannel strain sensor for monitoring physiological signals and energy collection (Fig. S21a). Figure 4h demonstrates a multiple-channel manipulator attached to a human hand to monitor finger movement, capturing five distinct gestures and their corresponding output responses. Each finger joint served as a source of current signals, with unique waveform features for each gesture, enabling accurate response recognition and classification. Additionally, due to the temperature difference (∼10 K) between the human body and the environment, each channel generated an output voltage of ∼35 mV. The customizable 3D-printed DHFG underscores its potential applications in more efficient TE conversion and multifunctional wearable applications.

### Highly customized DHFG to maximize thermal-energy utilization

Conventional thermocell fabrication methods, such as immersion, *in situ* polymerization and sol–gel processes [13–15], are typically inadequate for creating complex and irregular structures. These limitations lead to poor thermal-energy utilization and low conversion efficiency due to mismatched contact surfaces between hydrogel thermocells and complex heat sources. To address this challenge, the DHFG in a variety of shapes and configurations has been manufactured by using 3D-printing technology to precisely adapt to different application scenarios, maximize thermal energy and reduce assembly complexity and redundancy (Figs 1e and f).

**Figure 5.**
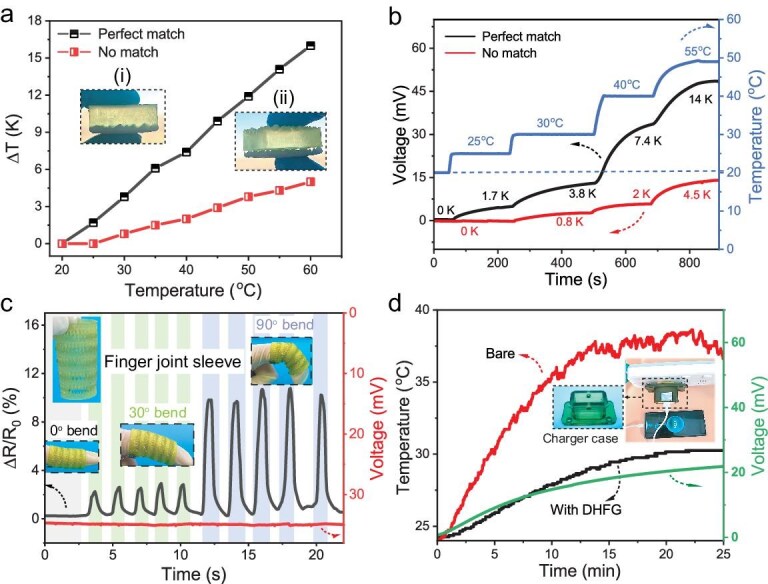
Performance evaluation of 3D-printed DHFG wearable thermocell under diverse conditions. (a) Temperature change and (b) output voltage of the DHF_0.2_G_3_ under the two conditions of (i) perfect match and (ii) mismatch at different temperatures of the irregular heat source. (c) Output voltage (right y-axis) and relative resistance (left y-axis) of the 3D-printed hollow DHF_0.2_G_3_ knuckle sleeve over time at different bending angles (0°, 30° and 60°), with electrodes at both ends contacting the skin and the external environment. The ∼10-K temperature difference was maintained by using a ∼36°C finger (pre-warmed) and ∼23°C ambient. (d) Temperature curve of the charger with and without the DHF_0.2_G_3_, together with the corresponding output voltage of the DHFG. The test was conducted by using a 22.5-W charger at an ambient temperature of ∼23°C.

Building on the 3D-printed wearable designs shown in Fig. 1e—including bracelets, insoles and headbands tailored to human body contours—we further optimized contact with complex heat sources. To simulate real-world thermal environments, we designed a material with an irregularly shaped interface (Fig. 1f). Using 3D printing, we engineered the DHF_0.2_G_3_ with one side precisely matching this irregular surface. When the irregular face of the DHF_0.2_G_3_ makes contact with the heat source, a ‘perfect match’ occurs; conversely, its smooth surface causes a ‘mismatch’ (Figs 1f and 5a). We then quantified the impact of these contact conditions on the Δ*T* and output voltage. As shown in Fig. 5a, the Δ*T* across the perfectly matched DHF_0.2_G_3_ increases more significantly with the heat-source temperature than in the mismatched configuration. Typically, as the ambient temperature is 20°C–22°C, the human bare-skin temperature is ∼25°C and, as the ambient temperature increases to 26°C–28°C, the bare-skin temperature rises to ∼30°C [47]. It can be observed that, at a heat-source temperature of 25°C, the planar DHFG cannot generate a Δ*T* for TE conversion due to insufficient contact with the complex heat source (Fig. 5a and b). In contrast, the microstructured DHF_0.2_G_3_, which perfectly conforms to the heat source, can generate a Δ*T* of 1.7 K. As the heat-source temperature increases to 30°C, the planar DHF_0.2_G_3_ begins to generate a small Δ*T* of 0.8 K, while the microstructured DHF_0.2_G_3_ exhibits a significantly higher Δ*T* of 3.2 K. Therefore, when harvesting human body heat, the microstructured DHF_0.2_G_3_ extends the usable ambient temperature range by 6 K. Moreover, at a heat-source temperature of 55°C, the output voltage of the microstructured DHF_0.2_G_3_ reaches ∼350% of that of the mismatched configuration (48.5 vs. 14 mV) (Fig. 5b), confirming that geometric matching directly boosts energy conversion.

As demonstrated in the wearable-sensing and thermal-management scenarios, the practical applications of the 3D-printed DHF_0.2_G_3_ extend well beyond conformal matching with heat sources, owing to its customizable architecture and multifunctional integration. Figure 5c showcases 3D-printed hollow DHF_0.2_G_3_ knuckle sleeves—designs that, like the body-adapted structures shown in Fig. 1e, fit snugly without tape. This design reduces assembly complexity while ensuring breathability. The output voltage generated by the temperature difference can serve as a self-powered information-conversion device, making this approach highly promising for applications in temperature detection and waste-heat collection. For these tests, DHF_0.2_G_3_ electrodes were configured with one end contacting skin and the other insulated with very high bond (VHB) tape to maintain a temperature gradient. When analysing the DHF_0.2_G_3_ knuckle sleeves at bending angles of 0°, 30° and 60°, we observed the relative resistance (left y-axis) increasing from 2% (30°) to 8% (90°), while the output voltage (right y-axis) remained stable at ∼35 mV (Δ*T* ∼10 K). This is because the output voltage is dependent solely on the temperature gradient and is unaffected by the shape of the DHF_0.2_G_3_. Additionally, bending the DHF_0.2_G_3_ relaxes the cross-linked polymer network, increasing its cross-sectional area and thus decreasing resistance while raising the relative resistance. These results confirm the stable TE properties and mechanical strength of the 3D-printed DHF_0.2_G_3_.

To further demonstrate versatility, we fabricated a 3D-printed DHF_0.2_G_3_ cover for a commercial phone charger (Fig. S21b and Fig. 5d). The DHF_0.2_G_3_ charger cover was applied to a commercial phone charger to address overheating risks and improve thermal management. A 2.0-mm-thick DHF_0.2_G_3_ was wrapped around the charger, with copper electrodes affixed to both sides. After 20 minutes of charging, the DHF_0.2_G_3_ reduced the temperature of the charger by 8 K with higher working security (Fig. 5d). Moreover, the DHF_0.2_G_3_ converted waste heat into electricity, generating a thermal voltage of ∼21 mV from a temperature difference of ∼6 K. It is noted that, as the water evaporates and is lost during the cooling process, the cooling effect and long-term functionality of the hydrogel for the charger are inevitably affected. To enhance the cooling effect and ensure long-term operation stability, it is possible to incorporate phase-change materials with high heat capacity into the hydrogel to improve the heat-storage capacity, and also dope some hydrophilic polymers and highly water-absorbent materials to improve the water retention of the hydrogel. Nevertheless, there may still be a long way to go before achieving this ultimate goal. Overall, this work highlights a transformative approach to designing advanced, adaptable thermocells tailored for a variety of complex heat-source scenarios, paving the way for innovations in wearable technologies and energy-harvesting systems.

## CONCLUSION

In this study, we successfully developed a stretchable high-fidelity 3D-printed DHFG with customizable microstructures by separating the 3D printing of DH eutectogel and the TE solvent-exchange step. The incorporation of nonvolatile DES and HEA—key components of the photopolymer ink precursor—facilitated the formation of a stable DHFG polymer network with high resolution. The addition of Gdm^+^ further enhanced the entropy difference of the redox couple and significantly improved the thermopower. The optimized 3D-printed DHFG exhibits excellent TE performance (3.5 mV K^−1^) and high structural resolution (down to 130 μm). Compared with the mismatched DHFG on the heat-source surface, the conformal design significantly extends the effective working temperature range by 6 K and increases the output power to ∼350% of that produced by the unmatched DHFG. Moreover, the microstructured DHFG demonstrated remarkable pressure sensitivity (0.35 kPa^−1^ below 1.0 kPa), which is nearly 4.4 times greater than those of its unstructured counterparts, enabling motion-sensing and health-monitoring capabilities. This innovative 3D-printing strategy not only maximizes thermal-energy utilization through precise geometric matching, but also streamlines the fabrication process of hydrogel thermocells, offering promising prospects for wearable TE devices and multifunctional sensing applications. This study also marks the first successful application of photocurable 3D printing in the fabrication of stretchable hydrogel thermocells, paving the way for more efficient TE materials and multifunctional wearable electronics, facilitating wider adaptability to a variety of complex heat sources.

## METHODS

### Materials

ChCl (98%), PEGDA_700_, AA (98%), *N*-hydroxyethyl acrylamide (HEA, AR), potassium ferrocyanide [K_4_Fe(CN)_6_] and GdmCl were purchased from Shanghai Macklin Biochemical Co., Ltd. TPO was purchased from Shanghai Aladdin Biochemical Technology Co., Ltd. Potassium ferricyanide [K_3_Fe(CN)_6_] was purchased from Tianjin heowns Biochemical Technology Co., Ltd. Gly was bought from Sigma-Aldrich. All materials were used without further purification. Deionized water was used in all experiments.

### Preparation of DESs

ChCl was mixed with hydrogen-bond donors, including AA and Gly, at a 1:2 molar ratio and stirred at 90°C for 4 hours until a homogeneous colorless liquid was formed. ChCl–AA (DES-1) and ChCl–Gly (DES-2) were selected at a 4:1 mass ratio as a deep eutectic solvent.

### Preparation of ink (DH)

The precursor solution for the ink, referred to as D_a_H_b_ (where a and b represent the mass ratios of the DES and HEA, respectively), was prepared using a one-pot method by directly mixing the AA, HEA, DES, PEGDA_700_ and TPO. Firstly, the DES and HEA were mixed at different mass ratios (3:1, 2:1, 1:1 and 1:2) in an ice bath for 30 minutes to form a transparent HEA/DES solution. Then, 0.1 wt% of PEGDA (based on the mass of the HEA and AA) was added to the DES solution and stirred. After the mixture was cooled in the ice bath, 1 wt% of TPO was introduced and the mixture was stirred until it formed a homogeneous precursor solution.

### 3D printing of the DH

All objects were printed by using a commercial DLP-3D printer (HALOT-SKY) from Shenzhen Nova Intelligent Technology Co., Ltd. The printing parameters were set as follows: layer exposure time of 5 s, motor lifting speed of 3 mm/s and six bottom exposure layers. The printer’s *X*/*Y* printing precision of 50 μm and *Z* precision of 10 μm were sufficient to meet the requirements for fabricating the DH structures. The entire printing process was performed without an additional protective atmosphere. After printing, the structures were washed with ethanol to remove the sacrificial printing solvent, photoinitiator and unreacted monomers/cross-linkers, followed by drying in an oven at 60°C for 30 minutes to obtain the final specimens. Eutectogels refers to systems using DES as the primary solvent. In this work, the initial 3D-printed gels are denoted as eutectogels (DH).

### Preparation of 3D-printed hydrogel thermocell (DHFG)

The 3D-printed object was fully immersed in mixed solutions of *x* M of K_3_Fe(CN)_6_/K_4_Fe(CN)_6_ and *y* M of GdmCl at 80°C for 3 hours to obtain the solvent-infused 3D-printed hydrogel thermocell (denoted as DHF*_x_*G*_y_*, where *x* = 0.05, 0.1, 0.2, 0.3 and 0.4 M; *y* = 0.5, 1.0, 2.0, 3.0 and 4.0 M). The resulting thermocell exhibited different tensile strengths and TE behaviors. Hydrogel thermocells are defined as systems in which water serves as the primary solvent and which contain functional redox couples. In this study, the DHFGs are obtained by immersing the DH in aqueous TE redox solutions, where solvent exchange introduces the necessary redox couples into the gel network.

## Supplementary Material

nwaf518_Supplemental_File
