# Supplementary material for: 3D printable shape-customized hydrogel thermocells
Source: Natl Sci Rev. 2025 Nov 19;13(11):nwaf518. doi: 10.1093/nsr/nwaf518 (PMC13215097; doi:10.1093/nsr/nwaf518)
Supplement: nwaf518_Supplemental_File [file nwaf518_supplemental_file.pdf]

Supplementary Information

3D printable shape-customized hydrogel thermocells

Lili Liu, Yiwen Bo, Ding Zhang*, Jiaqi Guo, Wenqi Fan, Ruohan Niu, Xin Guo, Xiaotian Jiang and Rujun Ma*

School of Materials Science and Engineering, Nankai University, Tianjin 300350, China

***Corresponding authors**. E-mails: malab@nankai.edu.cn; zhangding@nankai.edu.cn

Characterizations and Measurements

Scanning electron microscopy (SEM) images were obtained using a JSM-7800F JEOL microscope. Digital photos were taken using a Canon 5D Mark III camera. UV-vis absorbance spectra were recorded on a Specode 75 model spectrometer at a resolution of 4 cm^{-1} in the range of 3500-700 cm^{-1}. X-ray diffraction (XRD) data of DHFGs were measured on an X-ray diffractometer (Rigaku Smart Lab SE). The gel was structurally characterized using Fourier transform infrared (FT-IR) spectroscopy, covering the wavenumber range of 500 to 4000 cm^{-1}. The viscoelastic properties of the samples at different frequencies were analyzed using a DHR-2 rheometer (TA Instruments) with parallel plates (cylinders, 20 mm in diameter and 2 mm in height). Mechanical testing was performed using a universal mechanical testing machine (Instron 3342) at room temperature. Sample thickness was measured using a digital instrument. The tensile test was conducted at a speed of 100 $mm \cdot min^{-1}$. DHFGs were investigated for melting and freezing temperatures using differential scanning calorimetry (DSC 4000), scanning a temperature range from -100 °C to 25 °C (5 °C min^{-1}) under flowing N_2. Temperature measurement was performed using thermocouples and infrared camera (Fotric, 618C). Commercial platinum sheets are used as electrodes, and electrical signals were recorded using a Keithley 2450 digital multimeter.

Tensile measurement

The mechanical properties, including tensile strength and elongation at break, were determined through tensile tests on dog-bone-shaped samples using an Instron 3342 mechanical tester. Nominal tensile stress was defined as the force divided by the original cross-sectional area. The tensile strain (ε) was calculated as the elongation (ΔL) divided by the initial length (L_0) ($\varepsilon = \Delta L/L_0 \times 100\%$). Five hydrogel samples were measured for each test.

Calculation of the normalized output power density

In this study, the distance between the two electrodes is 2.0 cm. The output current-voltage curve was measured from 0 to the open circuit voltage (V_{oc}), corresponding to the short circuit current (I_{sc}) to 0 A. The instantaneous power is calculated by multiplying the corresponding output current and voltage values. For the normalized maximum power density ($P_{max}/(\Delta T)^2$), the calculation formula is as follows:

$$P_{max}/\Delta T^2 = V_{oc}I_{sc}/4\,\Delta T^2 \tag{S1}$$

Calculation of thermal energy conversion efficiency and Carnot relative efficiency

Thermal energy conversion efficiency (η) is a key parameter for evaluating the thermoelectric (TE) performance, which is defined as the ratio of the output power density (P_{max}) to the input power density (P_{heat}).

$$\eta = P_{max}/P_{heat} = P_{max} \cdot d/k \cdot \Delta T \tag{S2}$$

The energy conversion efficiency (η_r) of a heat engine relative to the Carnot efficiency limit is expressed as:

$$\eta_r = \eta/(\Delta T/T_{hot}) = P_{max} \cdot d \cdot T_{hot}/k \cdot \Delta T^2 \tag{S3}$$

where d is the electrode separation distance (2.0 cm), and κ is the thermal conductivity of DHFG.

Supplementary figures

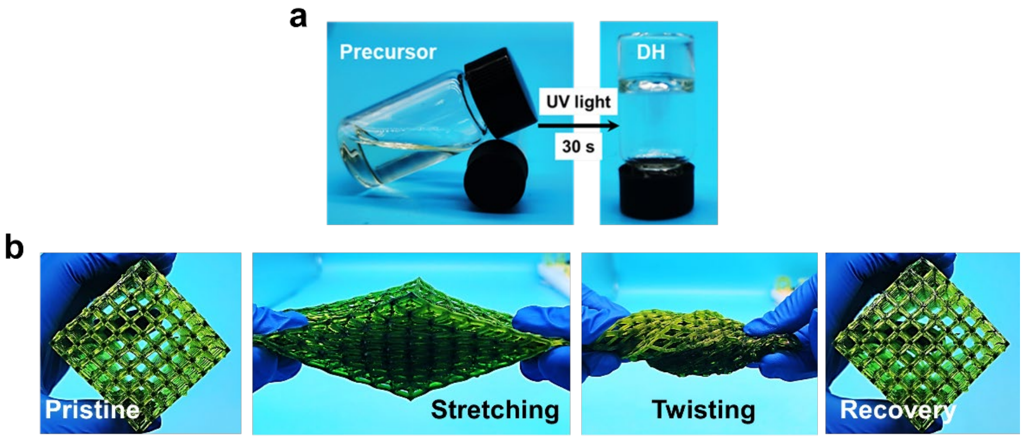

Figure S1. (**a**) Typical photo of DH after mixing all components of the precursor solution and photo-curing. (**b**) Mechanical behavior of DHFG, demonstrating its stretching and twisting capability.

As shown in **Figure S1a,** eutectogel (DH) can be formed after the uniform print prepolymer containing HEA and DES is irradiated by ultraviolet light for 30s. Moreover, the DHFG exhibits excellent elasticity and adapts well to bending, twisting and stretching (**Figure S1b**).

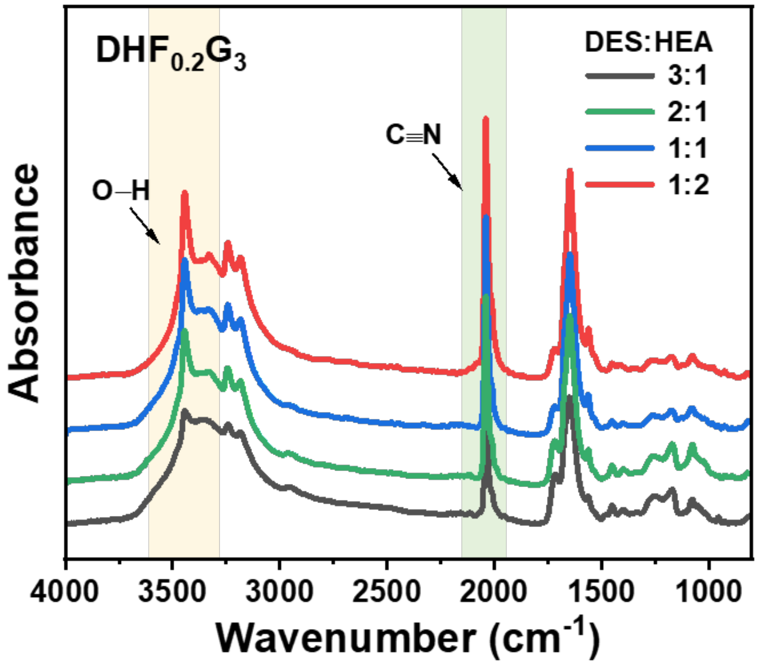

Figure S2. FTIR spectra of $DHF_{0.2}G_3$ with different ratios of DES and HEA.

The O-H stretching vibration peak in HEA gradually increases with the increase of the DES: HEA ratio as shown in **Figure S2**. The increase of HEA content amplifies the O-H tensile vibration peak, indicating that the interaction between HEA and DES is stronger. The C≡N stretching vibration observed at 2040 cm^{-1} for these samples indicates successful solvent exchange with 0.2 M $[Fe(CN)_6]^{4-/3-}$ -3.0 M GdmCl solution.

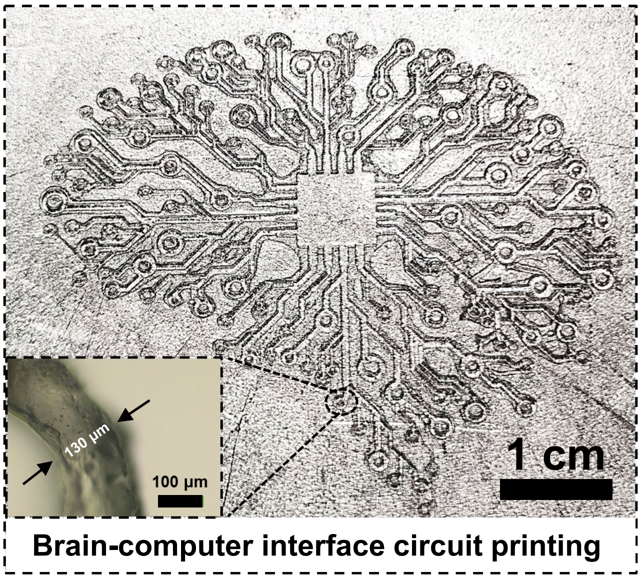

Figure S3. 3D printing of D_2H_1 ink-based brain computer interface microcircuit.

As shown in **Figure S3**, we successfully printed out a conceptual model of the brain-computer interface circuit printing with D_2H_1 ink. The microcircuit shows a width of 130 μm in the enlarged image, indicating that its accuracy can reach a size of 130 μm, which has great potential value in the field of wearable electronics.

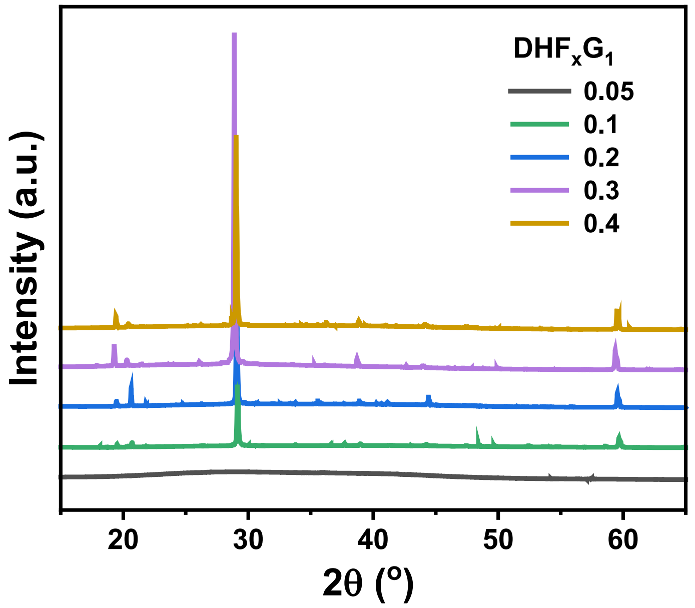

Figure S4. XRD patterns of DHF$_x$G$_1$ (DH represented D$_2$H$_1$, similar to the following).

The crystallinity of DHF$_x$G$_1$ gradually increases with the increase of x (**Figure S4**). The $[Fe(CN)_6]^{4-/3-}$ polarizes water molecules, forcing them to flow out between polymer chains, leading to aggregation or crystallization of the HEA/AA chains. As the concentration of $[Fe(CN)_6]^{4-/3-}$ increases, the "salting out" intensifies, resulting in a denser polymer chain and enhanced mechanical properties of DHF$_x$G$_1$.

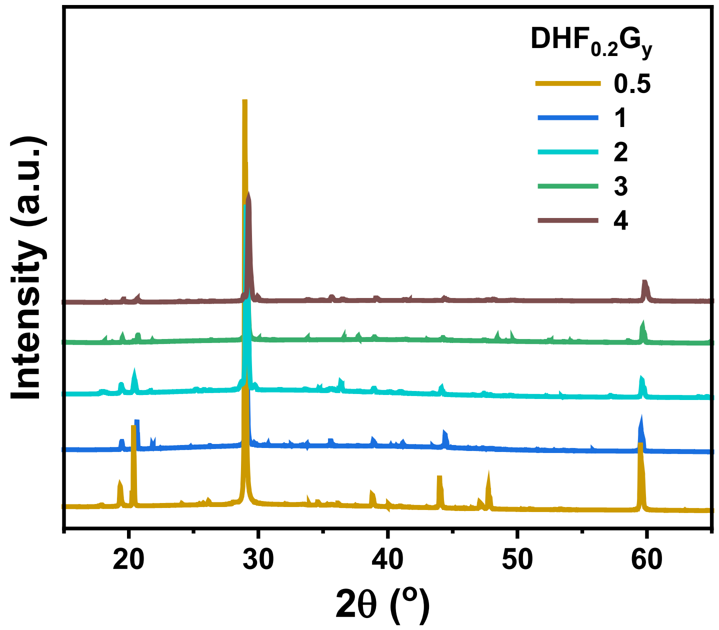

Figure S5. XRD patterns of $DHF_{0.2}G_y$, with y ranging from 0.5 to 4.0 M.

In contrast, for $DHF_{0.2}G_y$, the crystallinity decreases as y increases, as seen by the reduction in crystallization peak intensity (**Figure S5**). When GdmCl is added, Gdm^+ interacts with the HEA/AA chain, creating a "salt in" effect. Gdm^+ destroys the hydrated shell around the $[Fe(CN)_6]^{4-/3-}$ complex, restores the hydrogen bond between HEA/AA and its hydrated water, and reduces the crystallinity.

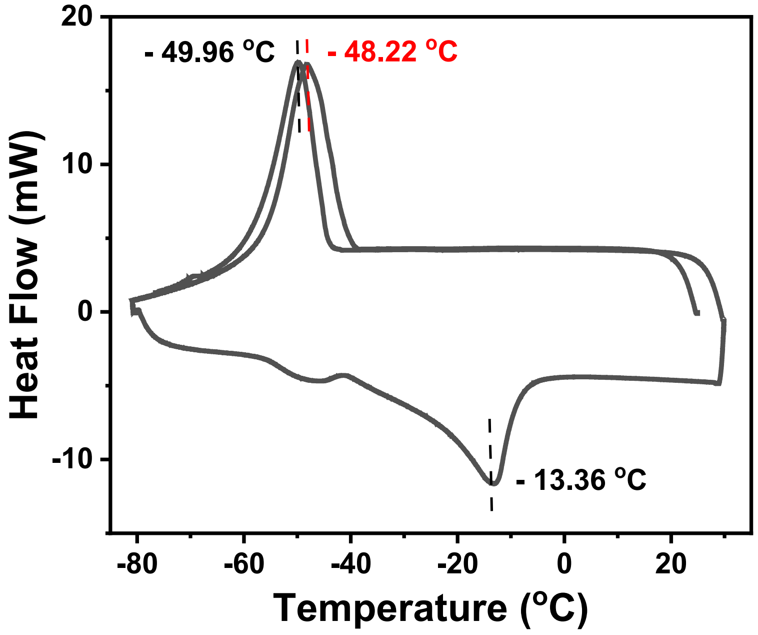

Figure S6. Differential scanning calorimetry (DSC) curves of $DHF_{0.2}G_3$.

Determined by differential scanning calorimetry (DSC), the freezing point of $DHF_{0.2}G_3$ was found to be - 48 ± 0.2 °C (**Figure S6**). This result indicates that a small amount of DES remained in the system after the thermoelectric solvent exchange process. It can be seen from this that, in addition to HEA interacting with water by the functional groups it contains that can form hydrogen bonds with water, the intense hydrogen bond interaction between the DES and H_2O molecules is the key factor. This intense interaction disrupts the original hydrogen bond network structure of water and then effectively inhibits the formation of ice crystals, enabling the system to exhibit excellent anti-freezing performance.

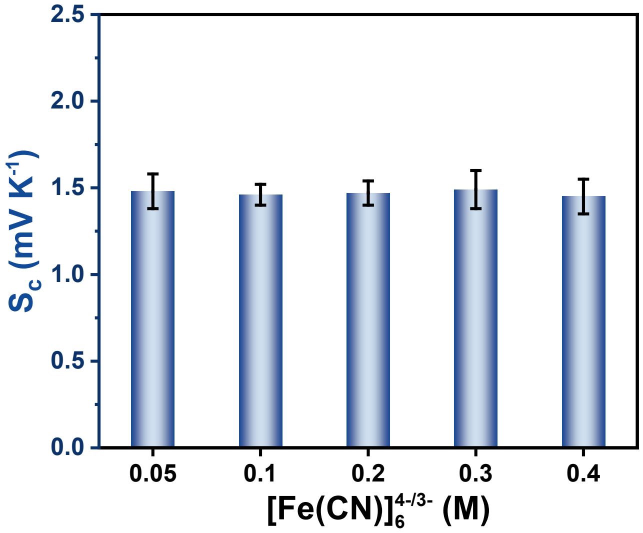

Figure S7. The S_c of the DHF_xG_0 with 0 M GdmCl, with x ranging from 0.05 to 0.4 M, where the x-axis represents the concentration (x) of the $[Fe(CN)_6]^{4-/3-}$.

The S_c of DHF_xG_0 were measured across different concentrations of x (0.05 to 0.4 M). As shown in **Figure S7**, the S_c remains relatively constant at ~1.48 mV K^{-1}, even as the concentration of x increases. This is likely because the activities of both redox ions increase proportionally, resulting in minimal net change in the relative entropy difference.

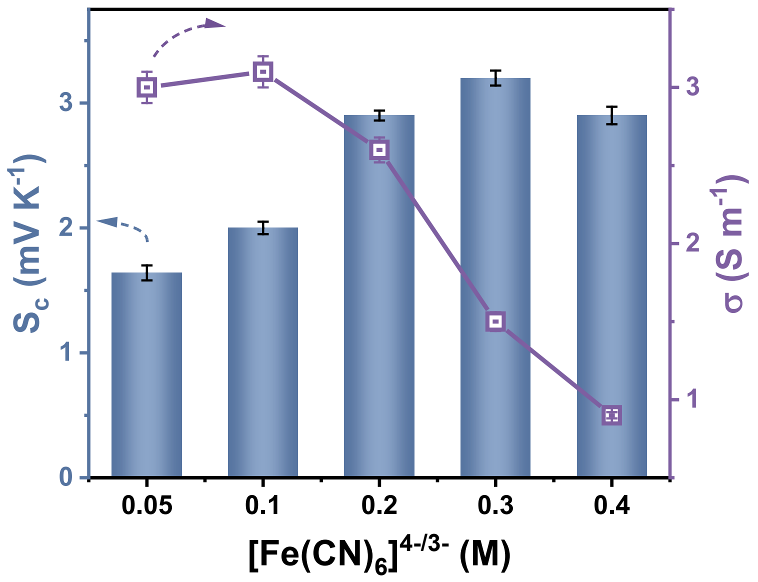

Figure S8. The S_c and σ of DHF_xG_1 with 1.0 M GdmCl (x ranging from 0.05 to 0.4 M), where the x-axis represents the concentration (x) of the $[Fe(CN)_6]^{4-/3-}$.

In contrast to the minimal S_c variation observed in DHF_xG_0 (**Figure S7**), the addition of GdmCl promotes the crystallization of $[Fe(CN)_6]^{4-}$ and improves the thermoelectric properties. The S_c of DHF_xG_1 is significantly higher than that of DHF_xG_0. $DHF_{0.4}G_1$ exhibits the highest S_c (3.2 mV K^{-1}) but the lowest σ (1.5 S m^{-1}) (**Figure S8**). As x increases, σ decreases from 3.1 S m^{-1} to 0.9 S m^{-1}. To further optimize the ratio, the TE performance, x was kept at 0.2 M while varying y. This improvement stems from Gdm^+-induced disruption of the $[Fe(CN)_6]^{4-}$ hydration shell, promoting its crystallization primarily at the cold side while increasing its thermal solubility. Upon heating, these crystals reversibly dissolve at the hot side, releasing additional $[Fe(CN)_6]^{4-}$ ions that enhance oxidation and amplify the interelectrode redox potential difference. This reversible process promotes ionic redistribution under a temperature difference and establishes the crystalline redox domains as dynamic ion reservoirs for sustained thermopower enhancement.

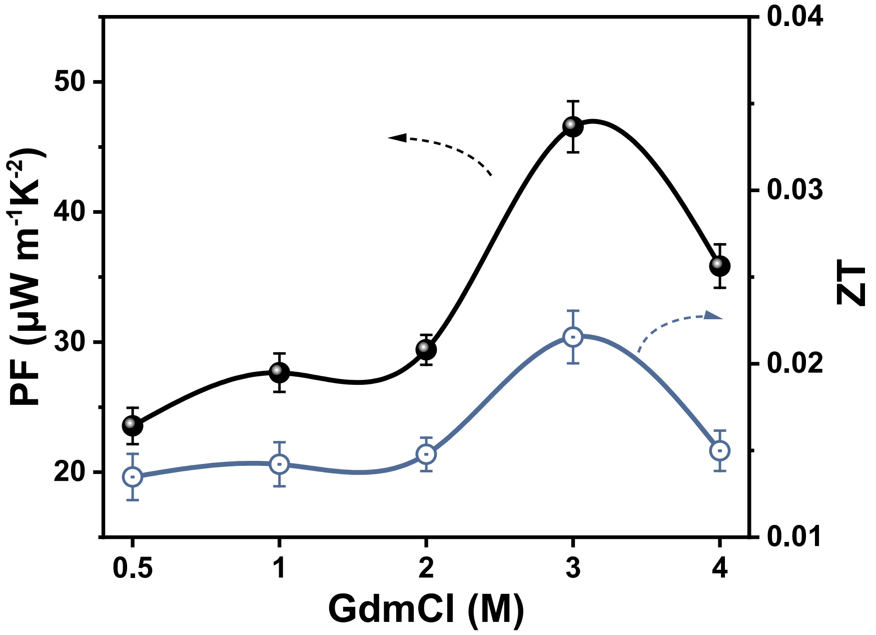

Figure S9. Power factor (PF) and figure of merit (ZT) of $DHF_{0.2}G_y$ with 0.2 M $[Fe(CN)_6]^{4-/3-}$ (y from 0.5 to 4.0 M), where the x-axis represents the concentration (y) of GdmCl.

Comparative analysis of power factor (PF) and figure of merit (ZT) showed that $DHF_{0.2}G_3$ outperformed other formulations, achieving a PF of 46 $\mu Wm^{-1}\cdot K^{-2}$ and a ZT of 0.022 (**Figure S9**).

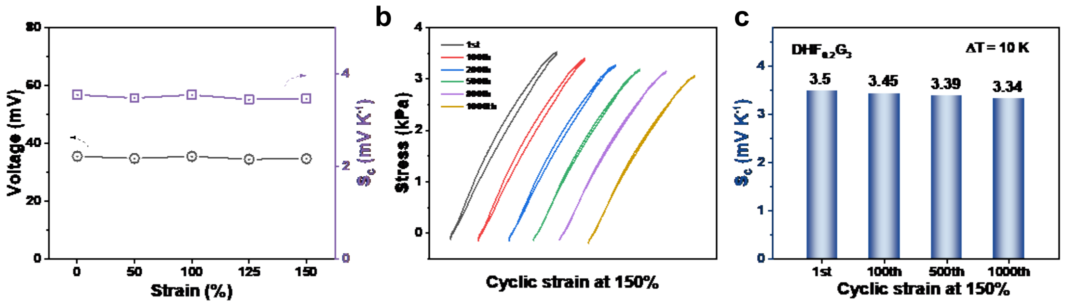

Figure S10. (**a**) Corresponding Voltage and S_c of $DHF_{0.2}G_3$ under different tensile strains at $\Delta T \sim 10$ K. (**b**) The stress-strain curves of the $DHF_{0.2}G_3$ were recorded at the 1st, 100th, 200th, 500th, 800th, and 1000th cycles under a 150% cyclic strain. (c) The output voltage of $DHF_{0.2}G_3$ at different cycles under 150% strain ($\Delta T = 10$ K).

To further confirm the stability and output performance of $DHF_{0.2}G_3$ under long-term cyclic bending and deformation, we performed systematic testing under various strain conditions and cycle numbers for evaluation. According to **Figure S10a**, for $DHF_{0.2}G_3$, both the S_c and σ maintain a relatively stable state, with values approximately at 3.5 mV K^{-1} and 35 mV, respectively, despite the increment in tensile strains. **Figure S10b** presents the cyclic stress-strain curves of $DHF_{0.2}G_3$ under 150% tensile strain, demonstrating its exceptional mechanical stability over 1000 cycles without significant hysteresis. Furthermore, after 1000 cyclic tensile strains, the S_c remains as high as 3.34 mV K^{-1}, corresponding to a high retention rate of 95.4% (**Figure S10b and S10c**). Therefore, long-term cyclic bending and deformation has little impact on the thermoelectric performance of DHFGs.

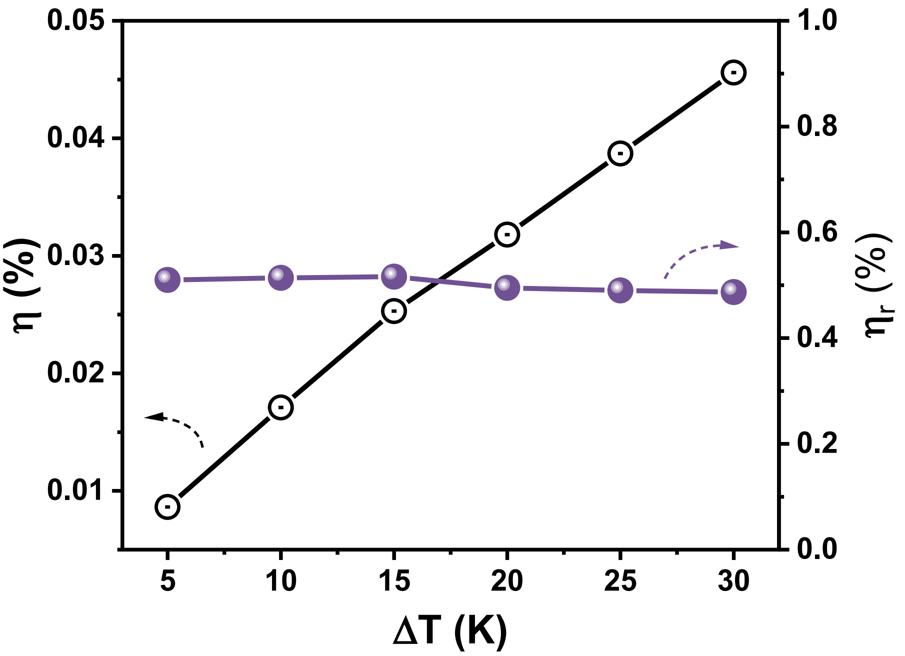

Figure S11. Thermal energy conversion efficiency of the $DHF_{0.2}G_3$ under different ΔT values.

Moreover, at ΔT ~ 30 K, the thermal energy conversion efficiency (η) and Carnot relative efficiency (η_r) of $DHF_{0.2}G_3$ reached 0.046% and 0.5%, respectively (**Figure S11**).

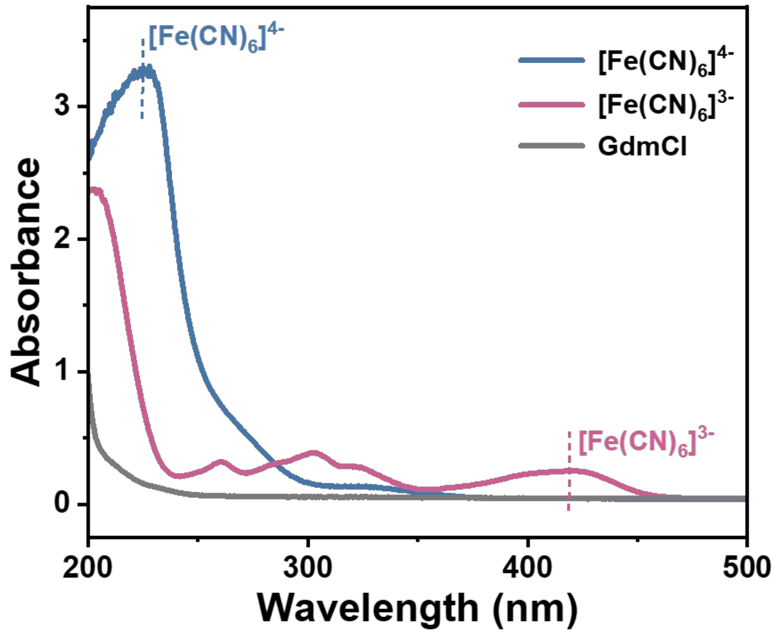

Figure S12. UV-vis spectra of $K_4[Fe(CN)_6]$, $K_3[Fe(CN)_6]$, and GdmCl.

Figure S12 clearly shows the significant differences in the UV-visible absorption spectra of the three substances. It directly reflected the differences between the optical absorption characteristics of $K_4[Fe(CN)_6]$, $K_3[Fe(CN)_6]$, and GdmCl, which were used to further analysis of the degree of their participation in the reaction.

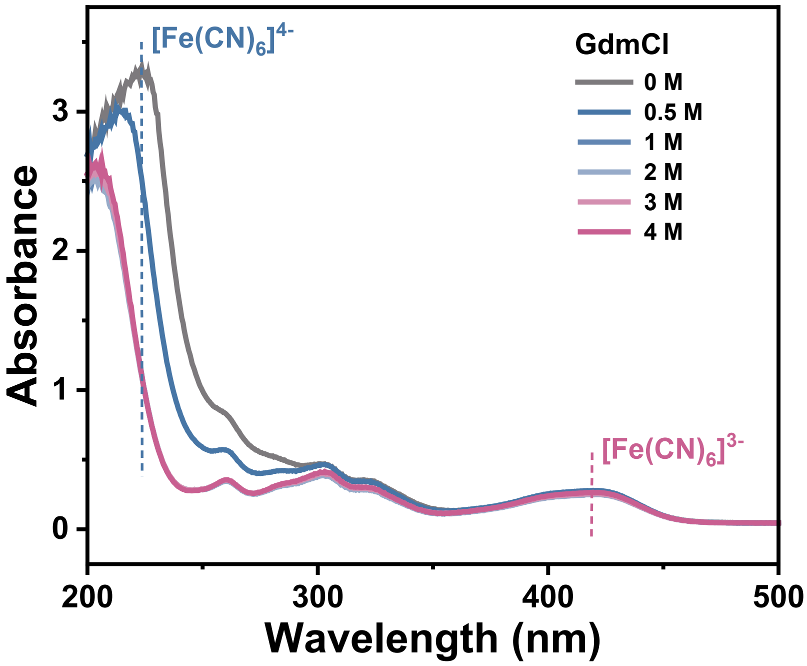

Figure S13. UV-vis spectra of 0.2 M $[Fe(CN)_6]^{4-/3-}$ with varying GdmCl concentrations (0, 0.5, 1.0, 2.0, 3.0, 4.0 M).

It can be observed from **Figure S13** that the addition of GdmCl leads to a significant decrease in the absorption peak of $[Fe(CN)_6]^{4-}$. In contrast, the absorption peak of $[Fe(CN)_6]^{3-}$ remains relatively stable. These findings imply that Gdm^+ exhibits a more pronounced interaction with $[Fe(CN)_6]^{4-}$.

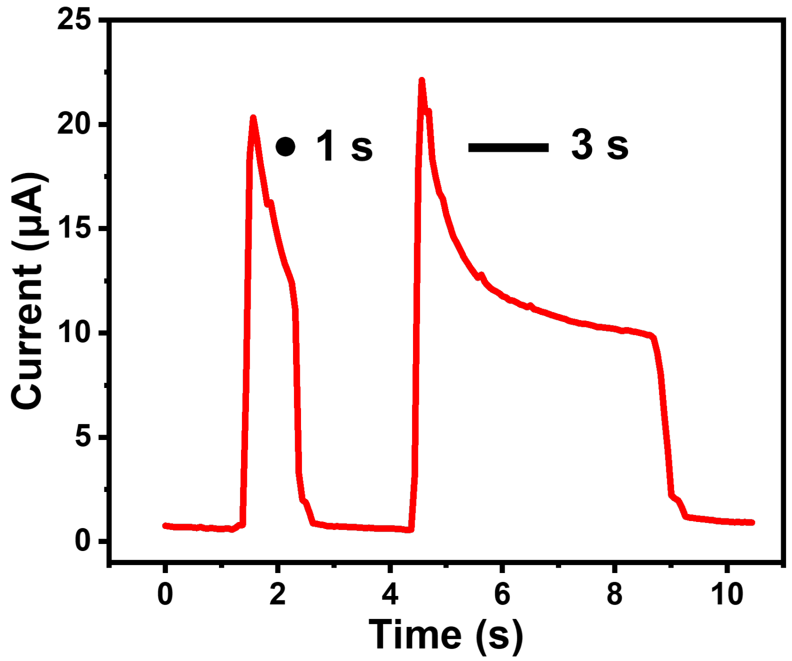

Figure S14. The point and line of finger temperature can be represented by different peak widths of $DHF_{0.2}G_3$ at 1 s and 3 s when the finger touches.

As shown in **Figure S14**, brief (1 s) and prolonged (3 s) of the finger to the $DHF_{0.2}G_3$ produced distinct signals corresponding to “dot” and “line” in Morse code, respectively. Electrical signals based on the thermogalvanic effect confirmed the feasibility of encoding “0” and “1” signals using this approach.

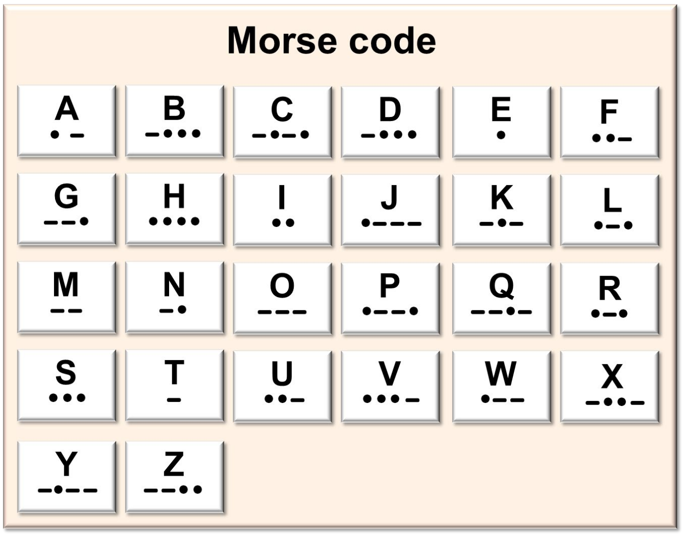

Figure S15. International Morse Code table.

Figure S15 shows the international Morse code table with the title "Morse Code". The table presents the Morse code corresponding to 26 letters (A-Z) in the form of squares, using the combination of dots (·) and dashes (-) to indicate the coding rules.

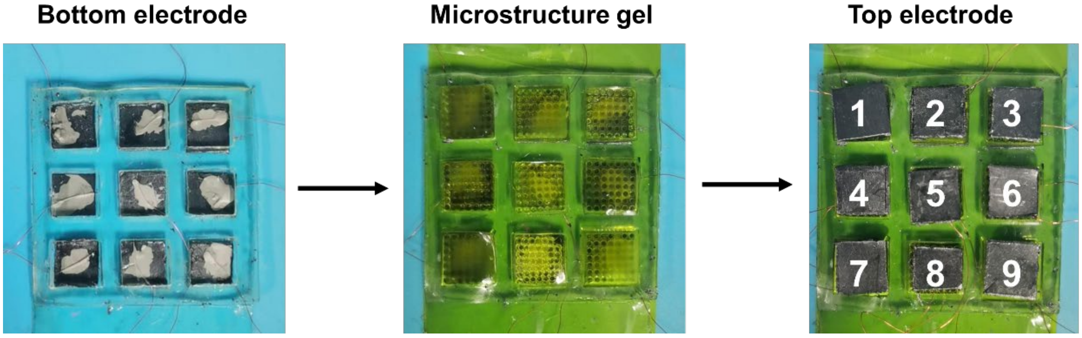

Figure S16. The preparation process of 3×3 pixel $DHF_{0.2}G_3$ sensor array.

To further explore spatial perception, we developed a 3×3 pixel $DHF_{0.2}G_3$ sensor array and attached it to the wrist to form a self-powered information conversion device for human-computer interaction (**Figure S16**). First of all, we 3D printed nine $DHF_{0.2}G_3$ with the same size and microstructure, cut 3M tape into nine hollow square structures with laser, placed the thermoelectric gel in it, extracted copper electrode on carbon paper as electrode material, and finally prepared the 3×3 pixel $DHF_{0.2}G_3$ sensor array by encapsulating both sides with PI film.

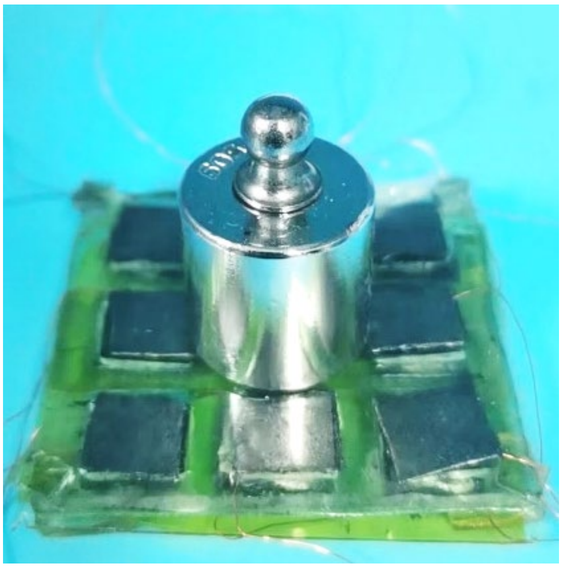

Figure S17. Schematic of placing weights in the center of the 3×3 pixel $DHF_{0.2}G_3$ sensor array.

A weight is placed in the middle of a 3×3 pixel $DHF_{0.2}G_3$ sensor array to measure various external tactile stimuli (**Figure S17**). The array is used to measure external tactile stimuli, applying pressure through weights to explore the response characteristics of the sensor array to different tactile stimuli.

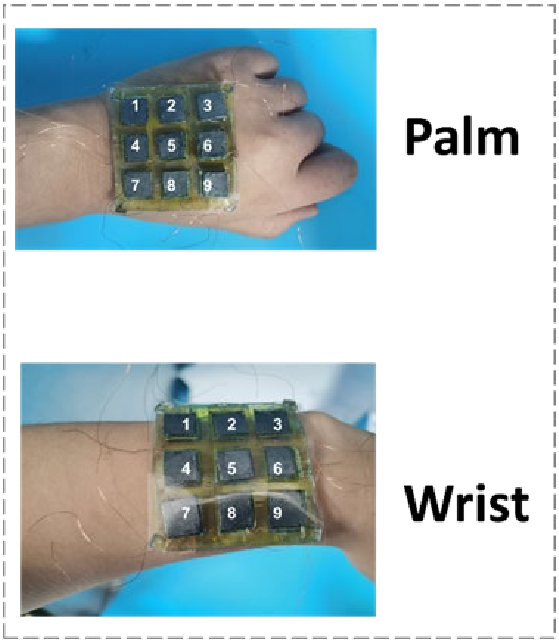

Figure S18. The optical image of the 3×3 pixel $DHF_{0.2}G_3$ sensor array device fixed on the back of the palm and wrist indicates its intrinsic flexibility and wearability.

A 3×3 pixel $DHF_{0.2}G_3$ sensor array was attached to the skin on the back of the palm and wrist to measure various external tactile stimuli (**Figure S18**).

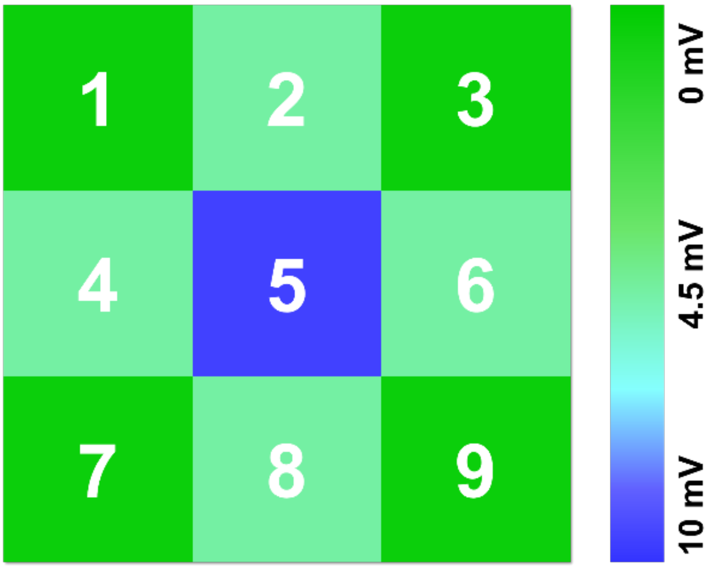

Figure S19. Voltage mapping image of the 3×3 sensing array system.

Figure S19 presents voltage mapping images showing pressure distribution. The spatial signal distribution of temperature corresponded to the position of the object, linked to the contact area between the object and the pixel. As shown in Figure S19, when pressure is applied to the middle of the array, the voltage mapping image of the 3×3 array device can be obtained. The signal measured by the center pixel was more substantial than the surrounding pixels, with the four surrounding pixels exhibiting nearly the same contact area as the object.

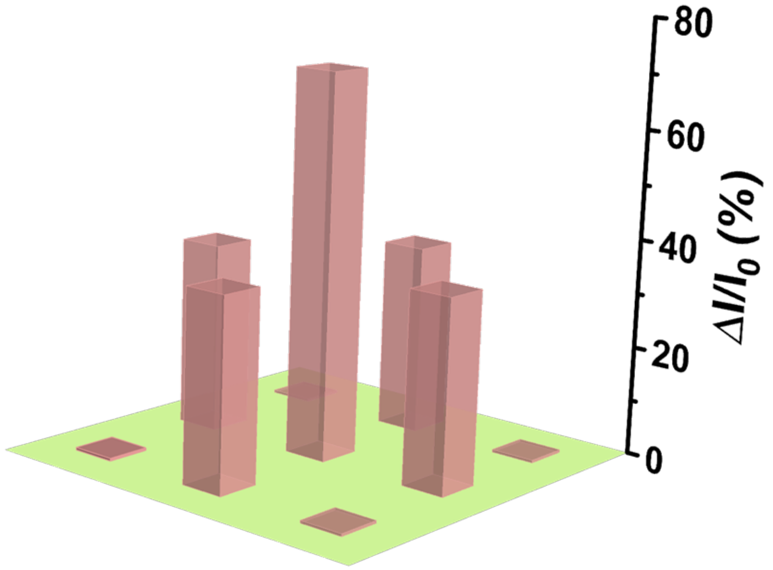

Figure S20. 3D bar charts depicting pressure distribution and the relative current when weight is centered on the 3×3 pixel $DHF_{0.2}G_3$ sensor array.

When a mass object touches the 3×3 pixel $DHF_{0.2}G_3$ sensor array, the stimulus can be quantitatively identified. An object is used to press the center of the sensor array in a natural environment to demonstrate its spatial pressure-sensing properties. The spatial tactile information collected by the $DHF_{0.2}G_3$ sensor array is shown in **Figure S20**. The spatial signal distribution of the pressure is consistent with the position of the heavy object, and the signal measured by the central pixel is stronger than the surrounding signal. This is related to the contact area between the object and the pixel.

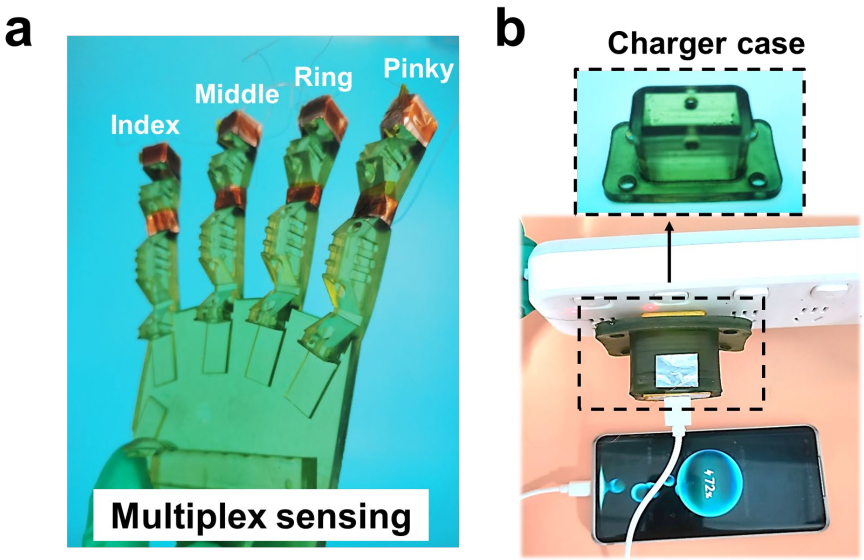

Figure S21. a,**b**, Photos of the 3D-printed (**a**) a multiple-channel $DHF_{0.2}G_3$ manipulator and (**b**) $DHF_{0.2}G_3$ phone charger cover flexible sensor.

The printed DHFG sensor array can be tailored for personalized human- machine interfaces and information encryption systems. As shown in **Figure S21a**, two positions of the fingers of the 3D printed $DHF_{0.2}G_3$ manipulator were selected with copper wires as electrodes, which were integrated into the multi-channel strain sensor for monitoring physiological signals and energy collection. A 3D-printed $DHF_{0.2}G_3$ phone charger cover was fabricated (**Figure S21b**). The $DHF_{0.2}G_3$ charger cover was applied to a commercial phone charger to address overheating risks and improve thermal management. A 2.0 mm thick $DHF_{0.2}G_3$ was wrapped around the charger, with copper electrodes affixed to both sides.
